# Management of Multiple Dental Trauma: Case Report with Eight-Year Follow-up

**DOI:** 10.22037/iej.v13i3.21090

**Published:** 2018

**Authors:** Alessandra Soares Ditzel, Ana Paula Tulio Manfron, Fernando Henrique Westphalen, Ulisses Xavier da Silva Neto, Alexandre Kowalczuck, Everdan Carneiro, Vânia Portela Ditzel Westphalen

**Affiliations:** a *Department of Oral Radiology, School of Life Sciences, Pontifícia Universidade Católica do Paraná. Curitiba, Paraná, Brazil;*; b *School of Dentistry, Faculdade Herrero.Curitiba, Paraná, Brazil;*; c *Department of Stomatology, School of Dentistry, Universidade Federal do Paraná, Curitiba, Paraná, Brazil;*; d *Department of Endodontics, School of Life Sciences, Pontifícia Universidade Católica do Paraná, Curitiba, Paraná, Brazil*

**Keywords:** Intrusion, Multiple Trauma, Tooth Fractures

## Abstract

This case report documents the clinical approach adopted for two maxillary incisors with intrusion and horizontal root fracture in the middle third after trauma. The proposed procedures involved maintaining pulp vitality and periodontal stability of the fractured teeth with 8 years of follow-up.

## Introduction

Dental intrusion is considered as one of the most severe dentoalveolar injuries, resulting in a displacement of the tooth in an axial direction [1-3] with the rate of occurrence being 2% in the permanent dentition [1]. The main sequelae are pulp necrosis, inflammatory external root resorption and replacement resorption [4-6]. Treatment of traumatically intruded teeth depends on root formation, if it is incomplete spontaneous recovery is expected, if it is complete, the surgical or orthodontic intervention is recommended [1, 7, 8].

In addition, another type of severe dental trauma, is the root fracture. The most common horizontal fractures occur in the middle third [9, 10], usually caused by frontal impact, comprised areas in the buccal and lingual regions, and involves dentin-pulp complex and cementum. In this way, pulp tissue and periodontal ligament cells are stimulated to promote healing process [11]. This occurs in permanent dentition in frequency of 0.5% to 7% [10, 12].

The treatment of root fracture depends on the pulp vitality, displacement of the fragments and the location/extension of the fracture line [11, 13]. The objective of this case report was to describe the treatment, evolution and eight-year follow-up of dental intrusion and horizontal fracture, at the same time.

## Case Report

**Figure 1:**
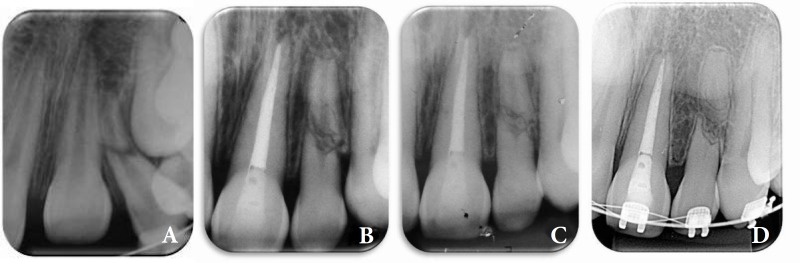
*A) *Initial periapical radiography demonstrating the intrusion of the left maxillary central incisor and the horizontal fractures in the middle third of the left maxillary lateral incisor; *B)* Periapical radiographs with 2 years follow-up; *C)* Periapical radiographs after 5 years follow-up; *D)* Periapical radiographs with 8 years follow-up

A 9-years-old female visited a dentoalveolar trauma clinic, 2 h after a bicycle accident. Clinical and radiographic examination revealed presence of a horizontal root fracture at the middle third of the left maxillary lateral incisor and 5 mm displacement in axial direction in the left maxillary central incisor. The patient received initial care after the accident including repositioning of the coronary portion of the left maxillary lateral incisor. Alveolar bone fracture was suspected and a rigid splint involving the maxillary incisors was used (Figure 1A).

Two weeks later during the second visit, surgical extrusion of the left maxillary central incisor was performed and antibiotic was prescribed (Amoxicillin/500 mg during 7 days). As the tooth apices were mature and spontaneous recovery might not occur, this procedure was chosen. The left maxillary central incisor showed negative responses to pulp sensibility test and dental splint was maintained for 3 months.

After 20 days, endodontic treatment of the intruded incisor was initiated and calcium hydroxide paste (Calen, SS White-Rio de Janeiro, RJ, Brazil) was placed in the root canal for a 4 weeks. This was followed by obturation and coronal restoration.

**Figure 2:**
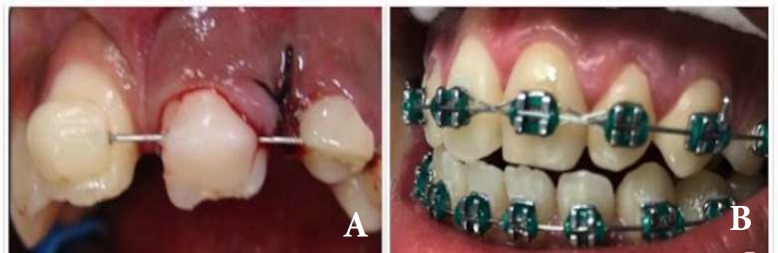
*A)* Clinical image after the surgical extrusion of the left maxillary central incisor; *B)* Clinical image after 8 years follow-up

Annual controls were performed. Clinical examination revealed normal soft and hard tissues, and left lateral incisor showed positive response to pulp sensitivity tests. During the first six months, monthly radiographic and clinical controls of all the involved teeth were performed. Clinical and radiographic follow-up, showed the stabilization of the horizontal root fracture in the middle third, normal soft and hard tissues, no evidences of root resorption in both teeth and a positive response to pulp sensibility tests of the left maxillary lateral incisor. After 8 years, the radiographic images suggested repairing of the left maxillary lateral incisor with deposition of mineralized tissue between the fragments (Figure 1B-D). Figures 2A and 2B showed the initial and final clinical images.

## Discussion

Dental intrusion might result in pulp necrosis in 45% of teeth with immature apices [5] and 100% in cases of mature apices [4]. Due to irreversible damage to cementum and periodontal ligament in this type of injury root resorption can happen [5, 6]. Wigen *et al.* [6] evaluated 51 teeth with dental intrusion, 20 had mature apices, of which only 5 maintained vital with posterior presence of pulp calcification. In addition, inflammatory root resorption was observed in seven of the 20 teeth with mature apices.

Lima *et al.* [4], reported the occurrence of 25% root resorption. In this case, after 8 years of follow-up, no radiographic signs were found compatible with root resorption in the left maxillary central incisor. Delays in root canal treatment increase the chances of developing root resorption in teeth with mature apices [14].

Certain events, such as sports injuries, violent incidents and road traffic accidents, may result in multiple dental injuries [15]. In this case, in addition to the intrusion of the left maxillary central incisor, a root fracture was also present.

The root fracture occurs as a consequence of an impact force in horizontal direction, often results in crown or crown-root fracture. This fracture can cause harmful consequences for dentin-pulp complex and periodontal tissues [16], however pulp vitality is better preserved in teeth that have undergone horizontal fractures than in teeth with dislocations and without root fractures [17].

The diameter of the apical foramen, condition of vascular support, number of cells available and degree of diastasis (separation between fragments) are fundamental to the prognosis [11]. Fractured teeth in the cervical third present the worse prognosis [16], due to the possibility of interposition of soft tissue between the fragments [17].

In this case, the fracture occurred in the middle third, that is favoring the maintenance of pulp vitality, corroborating with reported by Cvek *et al.* [9], who described a high survival rate in teeth with a middle third or apical fracture. Westphalen *et al.* [10] presented a case of pulp vitality in three fractured teeth after 13 years of follow-up. According to the authors the rapid reduction and immobilization of the fracture was instrumental in maintaining pulp vitality of the involved teeth.

Careful clinical and radiographic examination along with regular follow-up is essential, in order to minimize sequelae of dental trauma [7, 8, 18]. Periapical radiographs with different angulations are recommended as well as computed tomography, which provides 3D view [7, 8], with the possibility of more detailed observation of initial pathological signs [19]. However, it is important to highlight that computed tomography should be made to keep doses as low as possible [20]. 

In cases of dental intrusion and root fractures, clinical and radiographic controls should be performed after 6 months, 1 year and then annually [21]. In this case, it was not observed sequelae or dental complication after 8 years of follow-up. Although the literature described some complications with often poor prognosis of teeth that undergone dental intrusion follow surgical extrusion [1, 2], the presence of favorable conditions and appropriate protocols [21] increased the chance of successful treatment. The outcomes depends on the quality of dentist-patient relationship; dentists are responsible for the correct protocols application in different types of dental trauma [22] and the patient should follow the protocols and follow-up appointments [7, 21].

## Conclusion

The present case showed 8 years of stability, with aesthetic and dental function preserved. Long term of clinical and radiographic follow-up is important for the prognosis after trauma. The maintenance of the tooth, after traumatic episodes, has a direct impact in the patient's quality of life, restoring psychological and emotional states. 
